# Fluoride Metabolism in Pregnant Women: A Narrative Review of the Literature

**DOI:** 10.3390/metabo12040324

**Published:** 2022-04-02

**Authors:** Gina A. Castiblanco-Rubio, E. Angeles Martinez-Mier

**Affiliations:** Department of Cariology, Operative Dentistry and Dental Public Health, Indiana University School of Dentistry, 415 Lansing St., Indianapolis, IN 46202, USA; esmartin@iu.edu

**Keywords:** fluoride, fluoride metabolism, fluoride pharmacokinetics, pregnancy, pregnant, maternal-fetal fluoride exchange

## Abstract

Epidemiological studies use biomarkers of fluoride exposure in pregnant women as surrogate measures of fetal fluoride exposure; however, there is little understanding of how pregnancy affects fluoride metabolism and its biomarkers. This narrative review summarizes the changes of pregnancy that have the potential to impact fluoride’s absorption, distribution and excretion, and highlights the limited body of evidence on the topic. The physiologic systems that experience pregnancy-associated changes relevant to fluoride’s metabolism are the cardiovascular, renal, metabolic and gastrointestinal, as well bone and calcium metabolism and the body’s acid-base balance. The available evidence indicates that fluoride is found in the maternal plasma and urine, placenta, amniotic fluid and fetus. Although plasma and urinary fluoride vary across gestation, there is insufficient quality evidence to determine the direction or extent of such variation. Furthermore, there is no doubt that fluoride from maternal blood crosses the placenta and is absorbed and excreted by the fetus; however, the biological mechanisms behind this placental passage are unknown. Research on maternal and prenatal biomarkers of fluoride exposure would benefit from studies on how pregnancy-associated changes affect the metabolism of fluoride across gestation, the mechanisms for the intestinal absorption of fluoride in pregnant women, and the placental passage of fluoride.

## 1. Introduction

The fluoride ion is ubiquitous in the environment [1], constitutes a trace element of the human diet [2] and is of particular interest in dentistry and public health. Fluoride is considered a case of nutritional *hormesis*, the concept that the ingestion of small quantities has the opposite effect to ingestion of large quantities [3]. Fluoridation of community water [4] or salt for human consumption [5] has been established as an effective strategy for the prevention of dental caries, with stronger evidence supporting its effectiveness in children, and fewer studies in adults [6]. In contrast, fluoride exposure has also been associated with detrimental effects in human bones, teeth [7] and more recently neurodevelopment [8]. Considering that the prenatal period has recently been identified as a potential risk window for fluoride exposure, the challenge for those working towards achieving a balance between fluoride’s benefits and risks depends on finding the dose of exposure at which benefits can be maintained while minimizing side effects in vulnerable populations.

The epidemiological studies reporting associations between prenatal fluoride exposure and health outcomes in children have used biomarkers of fluoride exposure in pregnant women [9]. However, the use of these biomarkers has a major limitation: our narrow understanding of fluoride metabolism during pregnancy and how it may affect biomarker levels. Ideally, the choice of biomarkers of prenatal exposure should be informed by knowledge on how fluoride is absorbed, distributed and excreted by the mother and the fetus; unfortunately, that knowledge is limited. Responding to this knowledge gap, this narrative review aims to summarize the limited available evidence on the metabolism of fluoride in pregnant women (for thorough reviews on the metabolism of fluoride in the general population please refer to Whitford [10] and Buzalaf and Whitford [11]). An overview of the physiological changes of pregnancy will be presented, and those that potentially modify the metabolism of fluoride will be identified and summarized. Then, a systematic search and critical analysis of the literature on the absorption, distribution, and excretion of fluoride in pregnant women is presented, together with perspectives for future research.

## 2. Overview of Pregnancy and Summary of Changes with the Potential to Impact Fluoride Metabolism

During the first trimester of pregnancy, most physiologic changes are secondary to hormonal responses triggered after fertilization. One of the first changes observed in the first trimester occurs in the cardiovascular system: a dramatic increase in plasma volume, ~50% above nonpregnant values [12]. The new demand for oxygen increases the production of red blood cells by about 30%; however, they become diluted due to plasma volume increasing at a higher rate (low hematocrit due to hemodilution) [13]. These cardiovascular changes then lead to renal adaptations: the increased blood volume delivered to the kidneys raises the glomerular filtration rate by 40–60%. As a result, there is both an increase in urine flow and volume, and the filtration and excretion of water and solutes [14]. These cardiovascular and renal changes have the potential to dilute plasma and urinary fluoride levels.

As pregnancy progresses, the physical and physiological demands increase the body’s metabolic rate, leading to higher energy needs and nutritional requirements [15]. Additionally, the increased secretion of the hormones progesterone and relaxin, loosen smooth muscle and decrease the motility of the gastrointestinal tract, causing constipation, heartburn and reflux [16]. Pregnant women usually alleviate these symptoms through dietary modifications and drugs. Dietary modifications may include decreased consumption of triggers (highly acidic foods such as coffee, tomatoes and carbonated beverages) and an increase in the intake of healthier, less acidic alternatives [17]. On the other hand, the pharmacological management of gastrointestinal symptoms includes the use of antiacids (e.g., calcium carbonate, aluminum or magnesium hydroxide) and acid reducers (e.g., famotinide, ranitidine and omeprazole) [18]. Therefore, during pregnancy there is potential for a higher intake of fluoride [19], but also for decreased absorption through the stomach and small intestine, either because of decreased stomach acidity or increased consumption of calcium-containing supplements.

Around the second trimester, when the fetus’ presence becomes more evident, most physiological changes are secondary to fetal size. The gradual size-increase of the uterus pushes up the diaphragm up to 4 cm above its usual position, diminishing total lung capacity [16]. To compensate for the lower lung capacity, progesterone acts as a respiratory stimulant, increasing the volume of air inhaled per minute and leading to a state of *hyperventilation*—breathing faster and deeper [20]. The increased ventilation responds to the fetal demand for oxygen, but the increased exhalation of carbon dioxide (CO_2_) leads to disturbances in the body’s acid-base balance and the blood’s bicarbonate (HCO^−^_3_) buffering system: as more CO_2_ is lost, hydrogen (H^+^) ions are removed and blood acidity decreases, leading to *chronic respiratory alkalosis* [21]. To compensate for the decrease in blood H^+^, the kidneys excrete HCO^−^_3_ through the urine and retain H^+^ to maintain the blood’s pH at physiological levels [16]. This means that there may be a slightly more alkaline urine during pregnancy [22], a factor that has the potential to increase the urinary excretion of fluoride [11].

By the end of the second trimester, the intestinal absorption of calcium has doubled. This increased absorption allows for the buildup of maternal skeletal calcium stores to meet fetal demands during the third trimester [16]. Therefore, as pregnancy progresses, bone metabolism transitions from a state of predominantly maternal bone formation, increased bone density and calcium storage, to increased bone turnover for the transfer of calcium to the fetus towards the end of gestation [23]. The increased bone turnover of pregnancy has the potential to also release skeletal stores of fluoride.

**Figure 1:**
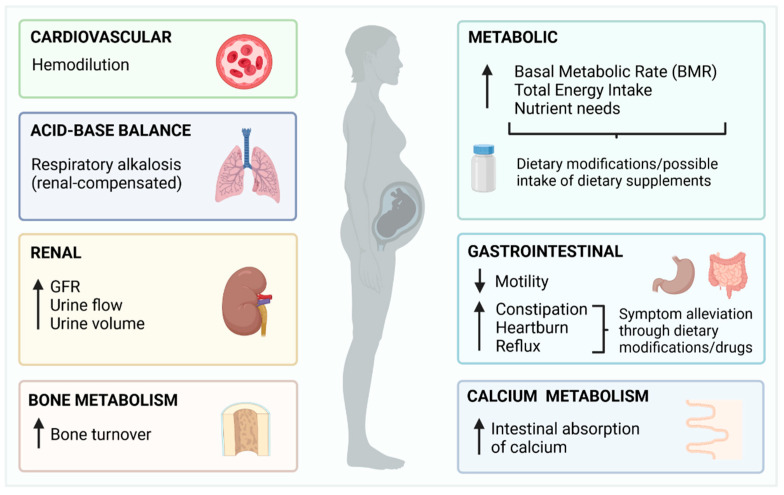
Summary of the physiological changes of pregnancy with potential to affect fluoride metabolism.

Overall, pregnancy induces changes in all major physiological systems [16], with some potentially affecting the absorption, distribution and excretion of fluoride. A summary of these physiological changes is depicted in Figure 1.

## 3. Available Evidence on the Absorption, Distribution, and Excretion of Fluoride in Pregnant Women

### 3.1. Literature Search

The literature search was conducted in two databases through the Ovid search interface: MEDLINE (1946 to 30 December 2021) and EMBASE (1974 to 30 December 2021), using a subject-heading approach. MEDLINE’s subject headings utilized were “Fluorides [Metabolism, Pharmacokinetics, Pharmacology, Physiology, Urine] AND Pregnancy or maternal-fetal exchange”. EMBASE’s identified subject headings were “Fluoride or fluoride ion AND fetomaternal transfusion”. The inclusion criteria were original investigations conducted in humans and reported in English language. The exclusion criteria were articles for which the full text was not available, articles which focused on a different topic (e.g., dental caries) or were grey literature. To remove duplicates, the citations from the papers that met inclusion and exclusion criteria from both databases, were exported to EndNote 20^®^. From the final articles retrieved from both databases (*n* = 29), additional references were identified by a manual search among the cited references (*n* = 12), adding to a total of 41 original articles critically reviewed for the following sections.

### 3.2. Fluoride Absorption in Pregnant Women

There is no data on the absorption of fluoride specifically gathered from pregnant women. In nonpregnant adults, and when ingested in the absence of inhibitors (such as food and calcium-containing products) [24], approximately 25% of the absorption of fluoride occurs in the stomach through a pH-dependent mechanism [25]. The remaining 75% of absorption occurs in the proximal small intestine through a pH-independent mechanism—via paracellular channels [26]. Whether these proportions and mechanisms are affected by the physiological adaptations and dietary modifications associated with pregnancy is unknown, and studies addressing these questions need to be conducted.

### 3.3. Distribution of Fluoride in Pregnant Women

#### 3.3.1. Maternal Blood

Fluoride in whole blood is the sum of ionic and nonionic fluoride. Ionic fluoride is the measure of significance for the health sciences and public health, and the one available to participate in biological reactions [11]. Under steady-state conditions (pH = 7.4 and normal hematocrit), ionic fluoride is asymmetrically distributed between plasma and blood cells in a 2:1 proportion [27]. Plasma is the central compartment for the distribution of fluoride. The levels of fluoride in plasma are not homeostatically regulated, and fluctuate depending on dietary and environmental exposure, exchange with soft and hard tissues, and urinary excretion. For this reason, plasma-fluoride levels have been used as a biomarker of present fluoride exposure [28]. In nonpregnant adults, fasting plasma-fluoride levels have been reported over a wide range, between 0.009 and 0.66 mg/L [28].

The first studies on fluoride levels in maternal plasma were motivated by the interest in the placental passage of fluoride, and the hypothesis that ingested fluoride could prenatally incorporate into developing enamel to make it resistant to dental caries [29]. As research evolved to conclude that the mechanism for the protective effect of fluoride on dental caries is not through prenatal incorporation into dental enamel but by topical exposure after tooth eruption [30], a gap arose in studies on maternal plasma fluoride levels (from the 1980s to the early 2000′s). Recently, there has been a renewed interest in this topic with studies using maternal plasma fluoride as a biomarker of prenatal exposure. A summary of available reports of plasma or serum fluoride levels in pregnant women [27,28,29,30,31] is displayed in Table 1. From this table, it can be inferred that reported plasma fluoride levels in pregnant women are within the range reported for nonpregnant adults [28]; however, two observations can be made: (1) as in nonpregnant people, maternal plasma/serum fluoride levels seem to be higher in areas with higher levels of fluoride in community fluoridation programs; and (2) fluoride levels seem to be lower compared to nonpregnant women and to decrease towards the end of gestation. The latter observation has been often interpreted as evidence of an association with increased fetal fluoride uptake at the time of fetal bone mineralization [31,32]. This assumption, however, ignores the physiologic hemodilution that occurs towards term. Interestingly, hemodilution peaks at 32 weeks [16], precisely when fluoride levels in pregnant women have been reported at their lowest. Furthermore, the changes in the acid-base balance that occur during pregnancy (such as physiologic hyperventilation or the metabolic acidosis associated with cases of gestational diabetes), lead to variations in blood pH that could potentially affect plasma or serum fluoride levels. To rule out the confounding effect of hemodilution in studies assessing plasma fluoride levels in pregnant women, future studies should control for this factor utilizing individual measures of hematocrit and consider gestational age and any condition involving disturbances in the body’s acid-base balance [27]. Whether the decrease in plasma and serum fluoride levels observed towards term is an artifact of changes in hemodynamics or the pregnant body’s acid-base balance, remains to be determined.

#### 3.3.2. Placenta

The placenta is a complex organ that constitutes an interface between the mother and fetus [16], and has metabolic, endocrine, immunologic and transport functions [32]. Fluoride has been measured in the placenta with high variability within and between samples [33]. Placental fluoride levels also vary depending on the sampling area, with higher concentrations in the placental periphery (probably as part of calcium precipitates that form in the periphery towards term) [34] and in placentas from women who had been supplemented with fluoride tablets or were living in fluoridated areas [34,35,36]. One study conducted in humans using radioactive fluoride, found that fluoride levels in pre-term placentas were lower than those of maternal plasma [37]. In contrast, studies in placentas collected at term, report higher levels than in the plasma [34,35,36]. The available evidence, therefore, suggests that fluoride is found in the placenta and its levels depend on exposure, placental sampling area, and gestational age.

#### 3.3.3. Placental Passage of Fluoride

There is placental passage of fluoride from the maternal to the fetal circulation. This conclusion comes both from studies on the relationship between maternal plasma (or serum) and cord blood [34,35,36,37,38,39,40,41,42,43,44,45,46,47,48,49,50,51], and studies on the metabolism of obstetrical anesthetics containing fluoride [52,53,54,55]. In the latter studies, there was evidence of fluoride in neonatal urine in levels that were proportional to the obstetrical anesthetic’s concentration, which provided further evidence on the transfer of fluoride from mother to fetus.

The transfer of fluoride to the fetus depends on maternal fluoride exposure levels. Higher concentrations of fluoride are found in the placenta, maternal plasma (or serum) and cord blood from women living in fluoridated areas [34,35,36]. In women who were supplemented with fluoride tablets at some point during their pregnancy, the concentration of fluoride in maternal plasma and cord blood increased as a function of the supplement’s concentration [45,48].

Currently, it is known that there are at least four different mechanisms of placental transport: passive diffusion, facilitated diffusion, active transport and pinocytosis [32]; nevertheless, the specific mechanism for the placental transport of fluoride is still unknown. The closest attempt to unraveling the mechanism for the placental transport of fluoride has been made by studies that measured levels of fluoride in cord blood and maternal plasma and have discussed the fetal-to-maternal plasma fluoride ratio (studies summarized in Table 2). In the summarized reports, the ratio between fetal/maternal fluoride concentration in plasma or serum varies from 0.25 to 1.66, with most studies reporting fluoride levels in cord blood lower than those of maternal blood. Fluoride levels in cord blood lower than those of maternal blood were generally assumed to be evidence of passive diffusion [29,36,39,40,41,42,43,44,46,47,50]. On the other hand, higher cord blood levels were considered evidence of an active placental role in maintaining fetal blood fluoride [36,38,45,49]. However, there are several limitations to the available studies that prevent reaching any conclusions regarding placental fluoride transport mechanisms. Most studies utilized analytical methods for the measurement of fluoride that are difficult to compare and did not provide details on gestational age or the sample’s collection protocol. For instance, most samples were collected during labor, or immediately after delivery, which are moments that are not representative of basal physiologic conditions, and in fact represent challenges for the body’s acid-base balance. Furthermore, there was little disclosure of possible confounders, such as the administration of intravenous fluids during labor and delivery (which can further dilute fluoride levels) or drugs that could potentially modify plasma or serum fluoride concentrations.

**Figure 2:**
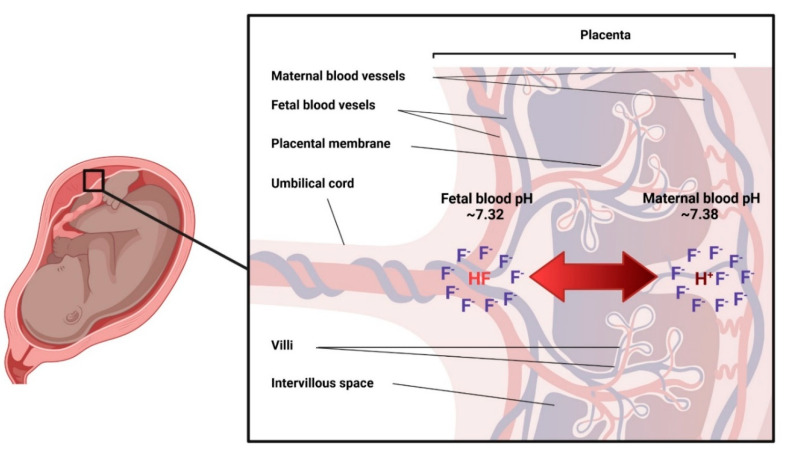
Hypothesis for the placental transfer of fluoride. Nonionized fluoride (HF, pKa = 3.4) diffuses passively through lipid bilayers and moves towards the most alkaline compartment. Under normal conditions, fluoride (F^−^) will remain slightly more concentrated in maternal blood due to the slightly more alkaline environment. However, any transient disturbance in the maternal or fetal blood pH has the potential to alter the direction of the movement of F^−^.

Although the studies summarized in Table 2 do not provide mechanistic insights into the placental transfer of fluoride, the considerable progress in research on both the metabolism of fluoride, and maternal-fetal pharmacokinetics, allows for the postulation of a hypothesis on the mechanism of the placental transfer of fluoride and a partial explanation for the variability in the fetal/maternal ratios of plasma fluoride observed. Nonionized fluoride in blood is a weak acid (Hydrogen Fluoride, HF) with pKa = 3.4, which diffuses passively through lipid bilayers and moves from the more acidic to the most alkaline compartment. Normally, maternal blood pH (7.38–7.42), is slightly more alkaline than fetal blood pH (7.32–7.38); therefore, under normal conditions fluoride will remain slightly more concentrated in maternal blood. However, any transient disturbance in the maternal or fetal blood pH will alter the movement of fluoride: a maternal blood pH that is slightly more acidic than fetal pH, will favor the movement of HF towards the fetal circulation. Under this scenario, once HF reaches the fetal circulation, it ionizes into H+ and F^-^, and the fluoride ion stays in the fetal compartment, a phenomenon that has been described in perinatal pharmacology as “fetal ion trapping” (Figure 2). This mechanistic hypothesis requires experimental evidence from in silico and in vivo studies; nevertheless, understanding that the placental passage of fluoride is potentially affected by transient disturbances of the maternal-fetal pH equilibrium provides a partial explanation for the high inter-individual variability of the ratio between fetal and maternal fluoride levels.

#### 3.3.4. Amniotic Fluid

The composition of amniotic fluid varies over the course of gestation. Early in pregnancy, when fetal skin has not reached full keratinization and allows exchange of water and solutes, the amniotic fluid’s composition is similar to maternal and fetal serum. In contrast, towards term, fetal urine output constitutes the major contribution to amniotic fluid [16]. Ionic fluoride has been reported in amniotic fluid at different moments of gestation and has been compared to maternal serum or plasma fluoride. In a sample of 47 women with a mean gestational age of 20.5 ± 2 weeks, the concentration of fluoride in amniotic fluid was highly correlated with that of maternal serum [31]. Two additional studies in women in their second trimester of pregnancy [48], and at 16–40 gestational weeks [44], report a proportion of fluoride in amniotic fluid lower to that found in maternal serum [44,48]. It is, therefore, reasonable to assume that the variations in fluoride concentration in amniotic fluid depend not only on the concentration of fluoride in maternal plasma, but also on the possible complex mechanisms of maternal-fetal transfer of fluoride (Figure 2).

#### 3.3.5. Fetus

The fetal uptake of fluoride is demonstrated by reports of varying levels of fluoride in fetal calcified [50,56,57,58,59,60,61] and soft tissues [62,63], even under similar intake and exposure conditions. Early investigations focused on the association between the concentration of fluoride in community water supplies and the concentration of fluoride in fetal hard tissues. From these studies, it can be concluded that the concentration of fluoride is higher in hard tissues from fetuses whose mothers were exposed to higher levels of fluoride through the public water supply [50,58,61]; and regardless of level of fluoride exposure (low or high), fluoride levels in fetal tissues increase with increasing gestational age [57,60]. Fluoride levels in fetal brains have also been reported to be higher in areas of high fluoride exposure (fluorosis endemic areas), compared with low-fluoride exposure (areas low in fluorosis) [62,63]. In calcified tissues, fluoride levels are reported to be higher in the femur, compared to the maxilla, mandible or developing teeth [58,59]; these differences may be explained by the degree of vascularization of the developing organ.

### 3.4. Urinary and fecal excretion of fluoride in pregnant women

In healthy adults, around 90% of any given amount of fluoride ingested is absorbed, and the non-absorbed portion (~10%) is excreted through the feces [10]. From the amount absorbed, about 60% will be excreted in the urine in less than 24 h [11]. In the limited number of reports from metabolic studies in pregnant women, it was observed that as well as in nonpregnant adults, most of the excretion of any given amount of ingested fluoride occurs through the urine, followed by the feces [64,65]. Other studies attempting to understand whether pregnancy affects the metabolism of fluoride, have compared urinary fluoride levels during gestation, with those from nonpregnant women [30,31,64,65,66,67,68,69]. A summary of available studies on urinary fluoride levels over the course of pregnancy is displayed in Table 3. Among the several limitations that prevent the comparison of the summarized studies, the use of spot urine samples with dilution adjustments (or no adjustment at all) is the major one, as only 24 h urine samples are appropriate to assess the urinary excretion of fluoride. Keeping these limitations in mind, from Table 3, two observations can be made: (1) urinary fluoride levels in pregnant women seem to be higher with increasing levels of fluoride intake or exposure; and (2) urinary fluoride levels do vary across pregnancy trimesters. In some cases, an observed decrease in urinary fluoride levels in spot urine towards term, has been discussed as evidence of fetal fluoride uptake [66,70]. Nevertheless, unless appropriate methods for the assessment of the urinary excretion of fluoride are used in future studies, whether urinary fluoride levels tend to increase or decrease towards term, and their association with fetal fluoride absorption, will remain unclear.

## 4. Conclusions

- Although pregnancy is a physiological state that affects all major systems (cardiovascular, renal, respiratory, gastrointestinal, bone metabolism and acid-base balance) with high potential to affect fluoride metabolism, the evidence on how these changes affect the intake, distribution, and excretion of fluoride, is limited in quantity and quality.
- Maternal plasma and urinary fluoride levels depend on fluoride exposure and vary across gestation, but there is not enough quality evidence to determine the direction (increase/decrease) of such variation.
- There is no doubt that fluoride from maternal blood crosses the placenta and is absorbed and excreted by the fetus. The biological mechanisms behind this transfer, are however, unknown.

In order to maximize fluoride’s benefits and minimize its risks in potentially vulnerable populations, it is important to understand the metabolism of fluoride during pregnancy, and the mechanisms behind the fetal uptake of fluoride. Historically, most studies on fluoride and pregnancy have focused on the fetus, with the pregnant woman as a passive carrier. Instead, future studies should focus on the complex physiological changes of the pregnant woman, and how these changes affect the metabolism of fluoride. Furthermore, this area may benefit from mechanistic studies to understand the intestinal absorption of fluoride in pregnant women and the placental passage of fluoride.

## Figures and Tables

**Table 1:** Summary of reports of plasma or serum fluoride levels in pregnant women.

Reference	Fluoride Source	Type of Sample	Analytical Method	n	Collection Time	Maternal F (mg/L)
Hanhijarvi [27], 1974	1 mg/L CWF	Plasma	ISE, microdiffusion	48	Not pregnant	0.023 ± 0.001
				149	Labor	0.018 ± 0.000
				67	0–10 months pregnant	0.018 ± 0.001
Hanhijarvi [28], 1981	1.2 mg/L CWF	Plasma	ISE, microdiffusion	12	Not pregnant	0.019 ± 0.006
				11	10 weeks	0.018 ± 0.000
				45	19 weeks	0.017 ± 0.005
				10	36 weeks	0.016 ± 0.003
				79	7 days after delivery	0.018 ± 0.006
Opydo-Szymaczek et al. [29], 2006	0.8 mg/L CWF	Plasma	ISE, known addition	31	28 weeks	0.062 ± 0.021
					33 weeks	0.071 ± 0.022
					Delivery	0.070 ± 0.026
Thomas et al. [30], 2016	250mg/kg CSF	Plasma	ISE, microdiffusion	220	13.5 ± 2.3 weeks	0.023 ± 0.022
				255	25.3 ± 2.4 weeks	0.019 ± 0.017
				152	34.5 ± 2.1 weeks	0.018 ± 0.017
Uyghurturk et al. [31], 2020	<0.7 mg/L CWF	Serum	ISE, microdiffusion	24	20.5 ± 2 weeks	0.011 ± 0.009
	>0.7 mg/L CWF	Serum	ISE, microdiffusion	24	20.5 ± 2 weeks	0.021 ± 0.015

ISE ion selective electrode. CWF community water fluoridation. CSF community salt fluoridation.

**Table 2:** Summary of studies reporting the ratio of fetal/maternal fluoride.

Reference	CWF (mg/L)	Type of Sample	Analytical Method	n	Collection Time	Maternal F (mg/L)	Fetal F^−^ (mg/L)	Ratio Fetal/Maternal	Correlation
Gedalia et al. [36], 1964	0.06	Not specified	Ashing and distillation	39	V	0.150 ± 0.060	0.160 ± 0.070	1.07	-
	0.60			30		0.230 ± 0.100	0.170 ± 0.050	0.74	-
Armstrong et al. [38], 1970	-	Plasma (venous)	Diffusion of Hydrogen F	16	C	0.100 ± 0.008	0.140 ± 0.013	1.40	-
		Plasma (arterial)		16		0.110 ± 0.011	0.140 ± 0.010	1.27	-
Shen & Taves [39], 1973	-	Serum	Morin-thorium reagent	16	V	0.017 ± 0.002	0.013 ± 0.002	0.76	0.86
Fry & Taves [40], 1973	-	Plasma	ISE, microdiffusion	7	V	-	-	0.25	-
Palahniuk & Cumming [41], 1975	-	Plasma	ISE, not specified	50	V	-	-	0.81	-
				41	L + V	-	-	0.63	-
				14	C	-	-	0.44	-
Weiss & Carlini [42], 1975	-	Serum	ISE, not specified	15	C	-	-	0.47	0.77
Louw et al. [43], 1984	-	Plasma	ISE, direct	10	C	0.270	0.250	0.92	0.94
Ron et al. [44], 1986	0.50	Plasma	ISE, direct	50	V	0.033 ± 0.003	0.028 ± 0.005	0.84	-
Caldera et al. [45], 1988	0.50	Plasma	Gas chromatography	46	V	0.022 ± 0.015	0.028 ± 0.021	1.26	-
Gupta et al. [46], 1993	-	Plasma	ISE, not specified	25	V	-	-	0.60	-
Malhotra et al. [47], 1993	1.20	Plasma	ISE, direct	25	V	0.250 ± 0.080	0.230 ± 0.840	0.92	0.97
Shimonovitz et al. [49], 1995	0.50	Serum	ISE, known-addition	22	V	0.018 ± 0.012	0.030 ± 0.015	1.66	-
Montherrat-Carret et al. [50], 1996	0.10	Serum	ISE, direct	5	17–25 weeks	0.034 ± 0.011	0.031 ± 0.011	0.91	-
Opydo-Szymaczek et al. [51], 2007	0.80	Plasma	ISE, known-addition	30	V	0.067 ± 0.023	0.055 ± 0.008	0.82	0.45

CWF community water fluoridation. V vaginal delivery. C cesarean delivery. ISE ion selective electrode. L labor.

**Table 3:** Summary of studies on urinary fluoride levels over the course of pregnancy.

Reference	Fluoride Source	Type of Sample	Analytical Method	Collection time		Sample Size	F concentration (mg/L)	Dilution Adjustment
Gedalia et al. [66], 1959	0.5–0.6 mg/L F CWF	Spot samples voided between 9:00 a.m. and 12:00 p.m.	Colorimetric after ashing and steam distillation	Pregnancy (months)	4	117	0.53	None
					5	80	0.43	
					6	91	0.34	
					7	89	0.28	
					8	81	0.22	
	0.5–0.6 mg/L F CWF	Spot samples voided between 9:00 a.m. and 12:00 p.m.	Colorimetric after ashing and steam distillation	Postpartum (months)	9	88	0.29	None
					1	55	0.39	
					2 & 3	64	0.49	
					4	45	0.50	
					8	32	0.23	
Maheshwari et al. [64], 1981	0.41 mg F/day, controlled diet high in animal protein	Pool of 3 days of 24 h samples	ISE, microdiffusion	Pregnancy (weeks)	20–40	6	0.62 ± 0.09	Not required
	0.27 mg F/day, controlled diet high in vegetable protein	Pool of 3 days of 24 h samples	ISE, microdiffusion	Pregnancy (weeks)	20–40	4	0.44 ± 0.07	Not required
	0.41 mg F/day, controlled diet high in animal protein	Pool of 3 days of 24 h samples	ISE, microdiffusion	Not pregnant	-	6	0.44 ± 0.04	Not required
	0.27 mg F/day, controlled diet high in vegetable protein	Pool of 3 days of 24 h samples	ISE, microdiffusion	Not pregnant	-	6	0.36 ± 0.05	Not required
Maheshwari et al. [65], 1983	1.35 mg F/day controlled diet	Pool of 3 days of 24 h samples	ISE, microdiffusion	Pregnancy (weeks)	10–20	6	0.95 ± 0.11	Not required
	1.40 mg F/day controlled diet	Pool of 3 days of 24 h samples	ISE, microdiffusion	Pregnancy (weeks)	30–40	5	1.03 ± 0.14	Not required
	1.28 mg F/day controlled diet	Pool of 3 days of 24 h samples	ISE, microdiffusion	Not pregnant	-	7	1.15 ± 0.24	Not required
Opydo-Szymackzek et al. [67], 2005	0.4–0.8 mg/L CWF	Spot morning urine (fasting)	ISE, direct	Pregnancy (weeks)	28–33	31	0.65 ± 0.316	None
							0.84 ± 0.352	
				Not pregnant	-	30	1.30 ± 0.301	
Thomas et al. [30], 2016	250 mg/kg in CSF	Spot morning urine (nonfasting)	ISE, microdiffusion	Pregnancy (weeks)	13.5 ± 2.3	436	0.92 ± 0.46	CRE
					25.3 ± 2.4	199	0.95 ± 0.47	
					34.5 ± 2.1	246	0.87 ± 0.48	
Till et al. [68], 2020	<0.3 mg/L CWF	Spot urine (collection time not specified)	ISE, microdiffusion	Pregnancy (weeks)	11.6 ± 1.6	541	0.31 ± 0.39	SG
					19.1 ± 2.4	507	0.39 ± 0.32	
					33.1 ± 1.5	475	0.48 ± 0.32	
	0.6–0.8 mg/L CWF	Spot urine (collection time not specified)	ISE, microdiffusion	Pregnancy (weeks)	11.6 ± 1.6	762	0.52 ± 0.46	SG
					19.1 ± 2.4	728	0.71 ± 0.47	
					33.1 ± 1.5	711	0.88 ± 0.55	
Uyghurturk et al. [31], 2020	<0.7 mg/L CWF	Spot urine (collection time not specified)	ISE, microdiffusion	Pregnancy (weeks)	20.5 ± 2.1	24	0.57 ± 0.35	SG
	>0.7 mg/L CWF					24	0.69 ± 0.34	
Castiblanco et al. [69], 2021	250 mg/kg CSF	Spot urine (collection time not specified)	ISE, microdiffusion	Pregnancy (weeks)	13.1 ± 2.1	135	0.82 ± 0.47	SG
					25.3 ± 2.1	101	0.83 ± 0.39	
					33.9 ± 2.6	71	0.86 ± 0.51	
			ISE, microdiffusion	Postpartum	12.2 ± 0.8	421	0.83 ± 0.37	

CWF community water fluoridation. CRE urinary creatinine. CSF community salt fluoridation. SG urinary specific gravity. ISE ion selective electrode.
